# Rolling Bearing Fault Diagnosis via Meta-BOHB Optimized CNN–Transformer Model and Time-Frequency Domain Analysis

**DOI:** 10.3390/s25226920

**Published:** 2025-11-12

**Authors:** Yikang Wang, He Jiang, Baoqi Tong, Shiwei Song

**Affiliations:** 1 School of Renewable Energy, Shenyang Institute of Engineering, Shenyang 110136, China; q1318217013@163.com (Y.W.); bqtong0912@gmail.com (B.T.); songshiwei0930@163.com (S.S.); 2 Key Laboratory of Regional Multi-Energy System Integration and Control, Shenyang 110136, China

**Keywords:** fault diagnosis, rolling bearings, vibration signal processing

## Abstract

Bearing fault diagnosis encounters limitations including insufficient accuracy, elevated model complexity, and demanding hyperparameter optimization. This research introduces a diagnostic framework combining variational mode decomposition (VMD) and fast Fourier transform (FFT) for extracting comprehensive temporal–spectral characteristics from vibration data. The methodology employs a hybrid deep learning architecture integrating convolutional neural networks (CNNs) with Transformers, where CNNs identify local features while Transformers capture extended dependencies. Meta-learning-enhanced Bayesian optimization and HyperBand (Meta-BOHB) is utilized for efficient hyperparameter selection. Evaluation on the Case Western Reserve University (CWRU) dataset using 5-fold cross-validation demonstrates a mean classification accuracy of 99.91% with exceptional stability (±0.08%). Comparative analysis reveals superior performance regarding precision, convergence rate, and loss metrics compared to existing approaches. Cross-dataset validation using Mechanical Fault Prevention Technology (MFPT) and Paderborn University (PU) datasets confirms robust generalization capabilities, achieving 100% and 98.75% accuracy within 5 and 7 iterations, respectively. Ablation studies validate the contribution of each component. Results demonstrate consistent performance across diverse experimental conditions, indicating significant potential for enhancing reliability and reducing operational costs in industrial fault diagnosis applications. The proposed method effectively addresses key challenges in bearing fault detection through advanced signal processing and optimized deep learning techniques.

## 1. Introduction

Rolling bearings, critical components of the transmission system, operate at high speeds under complex and variable conditions for extended periods. They frequently experience faults caused by impact, alternating loads and mechanical wear, which negatively affect mechanical system efficiency and result in economic losses [1]. Traditionally, experience-based fault diagnosis methods are often inadequate for early fault detection and real-time monitoring due to the large volume of data, complex feature sets, and inherent human subjectivity. Research indicates that approximately 40% of rotating machinery failures are attributed to bearings [2]. Therefore, the rapid and accurate diagnosis of faults in rolling bearings is critically important.

Conventional diagnostic approaches typically involve two key steps: processing sensor signals to extract embedded features, and then classifying faults using various algorithms. Among the common signal processing techniques employed are wavelet transforms [3], empirical mode decomposition (EMD) [4], spectral analysis [5], and the fast Fourier transform (FFT) [6]. For the classification stage, support vector machines (SVMs) [7] and artificial neural networks (ANNs) [8] are widely adopted. Despite the considerable success of these methodologies in bearing fault diagnosis, their dependence on manually engineered feature extraction poses significant challenges and limitations.

The evolution of deep learning technologies combined with enhanced computational capabilities has established new methodologies for automated fault identification systems. These advanced models possess the capability to extract critical patterns autonomously from raw sensor signals, substantially reducing dependence on conventional feature extraction procedures. Consequently, such methodologies have attracted considerable attention in mechanical bearing diagnostics research. Li et al. [9] developed an approach combining hierarchical symbolic dynamic entropy analysis with a tree-structured SVM classifier for fault identification. Meanwhile, Zheng et al. [10] proposed an adaptive meta-learning framework featuring variable input dimensions and attention mechanisms, demonstrating improved diagnostic performance under varying operational scenarios.

Significant contributions have emerged from various research groups. According to Guo et al. [11], intelligent fault diagnosis for machines with unlabeled data can be achieved through a novel deep convolutional transfer learning network. Wan et al. [12] implemented a Backpropagation Neural Network (BPNN) architecture enhanced through Quantum-behaved Particle Swarm Optimization (QPSO) techniques, incorporating Dempster-Shafer evidence theory fusion methodology for anomaly identification. Lin et al. [13] developed an innovative multi-resolution pooling-based domain adaptation framework, strengthening diagnostic reliability under diverse operating scenarios. Furthermore, Wen et al. [14,15,16] focused on adapting established 2D convolutional neural network (CNN) architectures, demonstrating their effectiveness in bearing defect classification tasks.

Building upon these foundations, recent research has increasingly explored hybrid architectures that combine the strengths of different deep learning paradigms. Liu et al. [17] introduced an adaptive frequency attention-based interpretable Transformer network, effectively addressing the challenge of few-shot fault diagnosis for rolling bearings. Subsequently, Lian et al. [18] proposed a residual attention-guided vision Transformer that leverages acoustic-vibration signal fusion for superior cross-domain fault diagnosis capabilities. In a parallel development, Wang et al. [19] designed WCFormer, an interpretable deep learning framework that demonstrates robust performance in heart sound analysis for automated cardiovascular disease diagnosis.

The increasing complexity of neural network architectures necessitates careful consideration of hyperparameter configuration. Researchers have increasingly adopted advanced optimization strategies to enhance diagnostic performance. Yao et al. [20] implemented Grey Wolf Optimizer (GWO) techniques to optimize Variational Mode Decomposition (VMD) decomposition parameters for hybrid transmission system diagnostics. Isham et al. [21] employed Data Envelopment Analysis (DEA) methods to configure VMD parameters, extracting multi-domain characteristics and improving fault identification precision. Wang et al. [22] utilized PSO algorithms to determine optimal VMD configurations, enabling automated parameter adjustment and achieving superior performance in bearing defect identification. These investigations emphasize how optimization algorithms contribute to improving neural network performance in industrial diagnostic tasks. Furthermore, emerging techniques, including dilated convolution layers and multi-head attention architectures, have demonstrated effectiveness in enhancing diagnostic precision, as evidenced by contemporary research [23]. Such advances illustrate the significance of optimization strategies in strengthening neural network capabilities for industrial diagnostic challenges. Notably, novel time-frequency feature extraction methods based on dynamic mode decomposition continue to push the boundaries of non-stationary signal analysis [24].

While deep learning and optimization methods have made substantial progress in bearing fault diagnosis, difficulties remain in processing non-stationary signals and managing noise interference within intricate operating environments. Moreover, the manual adjustment of hyperparameters proves both labor-intensive and susceptible to errors, which constrains real-world implementations.

Our proposed approach addresses these challenges by combining VMD and FFT techniques to decompose non-stationary signals reliably and derive frequency-domain characteristics, demonstrating particular effectiveness in complicated operational scenarios. Through integrating CNNs with Transformer architectures, we leverage the local pattern recognition abilities of CNNs and the extended dependency modeling capabilities of Transformers, achieving superior diagnostic accuracy and enhanced framework robustness. We further expedite hyperparameter optimization through the implementation of Meta-learning-enhanced Bayesian optimization and HyperBand (Meta-BOHB) optimizer [25], substantially decreasing both time investment and computational resources needed for manual parameter adjustment.

## 2. Transformer Model and VMD Algorithm

### 2.1. VMD

Konstantin Dragomiretskiy introduced Variational Mode Decomposition in 2014 [26] as an adaptive signal processing approach specifically developed for examining non-stationary and nonlinear signals. This completely non-recursive decomposition methodology adaptively decomposes intricate signals into a series of harmonic elements termed Intrinsic Mode Functions (IMFs). The core principle revolves around formulating and iteratively solving a constrained variational framework to establish optimal center frequencies and bandwidths for the IMFs. Throughout this procedure, each mode’s bandwidth undergoes iterative optimization to minimize overall bandwidth, facilitating efficient component separation and precise signal decomposition. The IMFs obtained through VMD demonstrate advantageous characteristics including sparsity, quasi-orthogonality, and narrowband properties [27,28].

VMD employs a constrained variational model, mathematically formulated as follows:

(1) $$\min\overset{K}{\underset{k=1}{\sum }}{\left|\frac{\partial }{\partial t}\left(\left(\delta \left(t\right)+\frac{j}{\pi t}\right)\cdot u_{k}\left(t\right)\cdot e^{-j\cdot \omega_{k}\cdot t}\right)\right|}^{2}$$

where $u_{k}$ denotes the kth IMF, $\omega_{k}$ represents its center frequency, $\delta \left(t\right)$ refers to the Dirac impulse function, and $e^{-j\cdot \omega_{k}\cdot t}$ describes the spectral shift in the IMF. The summation of all decomposed modes should recreate the initial signal $f\left(t\right)$:

(2) $$\overset{K}{\underset{k=1}{\sum }}u_{k}\left(t\right)=f\left(t\right)$$

For effective resolution of this constrained optimization challenge, a penalty parameter α along with a Lagrange coefficient $\lambda \left(t\right)$ are incorporated. This approach enables the conversion of the initial constrained formulation into an unconstrained optimization framework, represented through the augmented Lagrangian formulation:

(3) $$L\left(u_{k},\omega_{k},\lambda \right)=\alpha \overset{K}{\underset{k=1}{\sum }}{\left|\frac{\partial }{\partial t}\left(\left(\delta \left(t\right)+\frac{j}{\pi t}\right)\cdot u_{k}\left(t\right)\cdot e^{-j\omega_{k}\cdot t}\right)\right|}^{2}+{\left|f\left(t\right)-\overset{K}{\underset{k=1}{\sum }}u_{k}\left(t\right)\right|}^{2}+\lambda \left(t\right)\left(f\left(t\right)-\overset{K}{\underset{k=1}{\sum }}u_{k}\left(t\right)\right)$$

This constrained optimization problem is resolved using the Alternating Direction Method of Multipliers (ADMM) approach. The algorithm progressively adjusts the parameters of the decomposed modes $u_{k}\left(t\right)$, their central frequencies $\omega_{k}$, and the Lagrange multiplier $\lambda \left(t\right)$, minimizing the total bandwidth of each IMF. The iterative update expressions for $\omega_{k}$, $u_{k}\left(t\right)$, and $\lambda \left(t\right)$ are as follows:

1. Update of the center frequency $\omega_{k}$:

(4) $$\omega_{k}^{n+1}=\frac{\int_{0}^{\infty }\omega \cdot {\left|u_{k}\left(\omega \right)\right|}^{2}d\omega }{\int_{0}^{\infty }{\left|u_{k}\left(\omega \right)\right|}^{2}d\omega }$$

where $u_{k}\left(\omega \right)$ is the Fourier transform of $u_{k}\left(t\right)$, and the result represents the center frequency of the current mode.

2. Update of the modal component $u_{k}\left(t\right)$:

(5) $$u_{k}^{n+1}\left(\omega \right)=\frac{\hat{f}\left(\omega \right)-\sum_{i\neq k}{\hat{u}}_{i}\left(\omega \right)+\hat{\lambda }\left(\omega \right)}{2\left(1+2\alpha {\left(\omega -\omega_{k}\right)}^{2}\right)}$$

where $\hat{f}\left(\omega \right)$ is the Fourier transform of the original signal $f\left(t\right)$, ${\hat{u}}_{i}\left(\omega \right)$ represents the modal components excluding $u_{k}\left(\omega \right)$, and $\hat{\lambda }\left(\omega \right)$ is the Fourier transform of the Lagrange multiplier $\lambda \left(t\right)$.

3. Update of the Lagrange multiplier $\lambda \left(t\right)$:

(6) $$\lambda^{n+1}\left(t\right)=\lambda^{n}\left(t\right)+\tau \cdot \left(f\left(t\right)-\overset{K}{\underset{k=1}{\sum }}u_{k}^{n+1}\left(t\right)\right)$$

where τ is the step size used in the ADMM algorithm, and the Lagrange multiplier ensures the enforcement of the reconstruction constraint.

The repetitive cycle proceeds continuously until satisfying a pre-established halt condition:

(7) $$\sum_{k}\frac{{\left|u_{k}^{n+1}(t)-u_{k}^{n}(t)\right|}^{2}}{{\left|u_{k}^{n}(t)\right|}^{2}}<\epsilon$$

where ϵ represents the precision threshold for the convergence criterion. Via this methodology, VMD separates the signal into K modal elements with limited bandwidth, each possessing a unique central frequency $\omega_{k}$.

In contrast to conventional recursive decomposition techniques, VMD reformulates the mode extraction task as a non-recursive variational optimization problem. It iteratively refines both the modal components and their respective center frequencies. By employing the ADMM to solve this variational model, VMD effectively minimizes the aggregate bandwidth of all modes. This theoretical framework endows VMD with enhanced robustness to noise compared to recursive decomposition approaches [29]. Moreover, VMD demonstrates a strong capability to distinguish closely spaced harmonic components, thereby enabling precise extraction of fault-related information from complex signals [30].

### 2.2. Transformer

Transformer encoder, initially introduced by Vaswani et al. in 2017 [31], has demonstrated effectiveness as a robust technique beyond natural language processing, extending to domains including fault diagnosis, where the capability to model extended dependencies and intricate patterns within temporal data proves essential. In contrast to conventional approaches such as recurrent neural networks (RNNs), Transformer encoder employs an attention-based framework that handles all input elements simultaneously. This proves especially valuable for fault diagnosis applications, where signals typically demonstrate prolonged correlations, interference, and time-varying properties.

The self-attention framework evaluates correlations among various temporal positions within the input data stream. For fault detection applications, this encompasses sensor measurements or vibration data collected across temporal intervals. The Scaled Dot-Product Attention mechanism implemented in Transformer architecture is formulated as:

(8) $$Attention(Q,K,V)=softmax\left(\frac{QK^{T}}{\sqrt{d_{k}}}\right)V$$

where Q (queries), K (keys), and V (values) represent linear projections of the original input features. Within fault diagnostic systems, Q, K, and V encode distinct aspects of sensor data or related diagnostic indicators. The softmax operation normalizes the attention weights, enabling the architecture to prioritize critical temporal positions within the data stream, particularly abrupt variations or irregularities that signal potential failures.

To improve the modeling of complex dependencies, Transformer architecture employs multi-head attention mechanisms, integrating multiple attention components operating in parallel, where each component focuses on distinct signal features. The mathematical formulation for multi-head attention is expressed as:

(9) $$MultiHead(Q,K,V)=Concat(head_{1},\ldots ,head_{h})W^{O}$$

where each head $head_{i}$ is calculated as:

(10) $$head_{i}=Attention(QW_{i}^{Q},KW_{i}^{K},VW_{i}^{V})$$

where $W_{i}^{Q}$, $W_{i}^{K}$, and $W_{i}^{V}$ are learned projection matrices that help the model attend to different features of the input sequence, such as repetitive patterns or spikes in sensor data, which are often associated with machinery faults.

Following multi-head attention processing, Transformer encoder routes the data through a position-wise feed-forward layer, introducing non-linearity while strengthening the model’s capacity to derive significant features from diagnostic signals. The feed-forward layer is formulated as:

(11) $$FFN(x)=\max(0,xW_{1}+b_{1})W_{2}+b_{2}$$

where $W_{1}$, $W_{2}$, $b_{1}$, and $b_{2}$ represent trainable parameters. This architecture enables the model to identify intricate, non-linear associations among input features, which proves essential in fault diagnosis where incipient faults frequently appear as minor signal fluctuations.

One of the key challenges in fault diagnosis is dealing with sequential data while retaining positional information. Since Transformer encoder processes data in parallel, positional encodings are introduced to encode the relative positions of data points in the input sequence, ensuring that the model understands the temporal structure of the signals. The positional encoding is defined as:

(12) $$\left\{\begin{array}{l} PE_{\left(pos,2i\right)}=\sin\left(\frac{pos}{10000^{2i/d_{\mathrm{mod}el}}}\right)\\ PE_{\left(pos,2i+1\right)}=\cos\left(\frac{pos}{10000^{2i/d_{\mathrm{mod}el}}}\right) \end{array}\right.$$

where pos is the position and i is the dimension index. This is particularly important in fault diagnosis, where the temporal order of signal readings is essential for detecting abnormal patterns.

**Figure 1:**
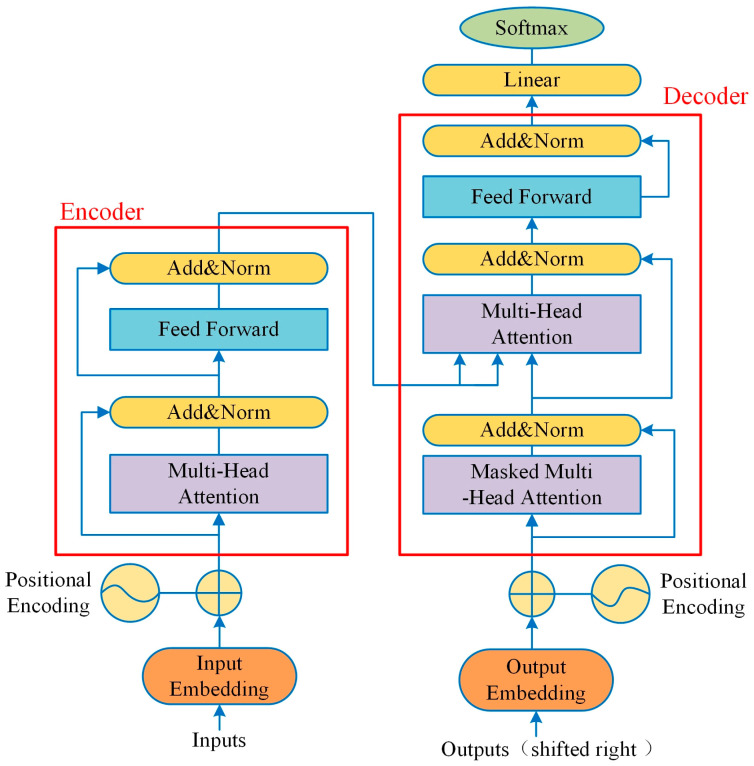
The structure of Transformer model.

Transformer encoder proves highly effective for fault diagnosis applications via attention mechanisms, multi-head processing, and position-wise feed-forward layers. As depicted in Figure 1, its architecture effectively captures long-range dependencies and complex signal relationships, allowing for the accurate identification of faults and early-stage anomalies—even in noisy and non-stationary signals. This makes Transformer encoder a highly effective tool in predictive maintenance and machinery health monitoring.

## 3. Meta-Learning Enhanced Optimization Techniques

Meta-learning serves an essential function in enhancing both the effectiveness and adaptability of model configuration processes. Conventional hyperparameter adjustment methods typically require extensive computational time and are susceptible to errors, which restricts the operational performance and real-world deployment of neural network architectures. Through the implementation of meta-learning approaches, optimization techniques are acquired at an elevated conceptual level, thereby expediting the hyperparameter identification procedure.

### 3.1. Bayesian Optimization and Hyperband

Bayesian Optimization and HyperBand (BOHB) is an advanced hyperparameter optimization technique that synergistically combines Bayesian Optimization (BO) [32], known for its probabilistic surrogate modeling, with HyperBand (HB) [33], recognized for its efficient resource allocation through early stopping. The primary objective of BOHB is to optimize hyperparameters λ by minimizing a black-box objective function f(λ), typically representing validation loss:

(13) $$\lambda^{*}=arg\underset{\lambda \in \Lambda }{\min}f(\lambda )$$

where Λ represents the hyperparameter search space. BO utilizes a probabilistic model p(f|λ), typically a Gaussian Process (GP) or a Tree-structured Parzen Estimator (TPE), to estimate the objective function using available observations $D={\left\{(\lambda_{i},f(\lambda_{i}))\right\}}_{i=1}^{n}$. The Expected Improvement (EI) selection function dictates the choice of subsequent hyperparameter configurations for assessment:

(14) $$EI(\lambda )=\int_{-\infty }^{f_{\mathrm{best}}}\left(f_{\mathrm{best}}-f\right)p(f|\lambda )df$$

where $f_{\mathrm{best}}$ is the best-observed value. By integrating HyperBand, BOHB efficiently allocates computational resources through successive halving, focusing on the most promising hyperparameter configurations while terminating early the less promising ones. This approach achieves a harmonious balance in investigating and utilizing the hyperparameter range, thereby boosting the efficiency and precision of the optimization procedure.

The key innovation in BOHB lies in the utilization of the TPE [34], which models the densities of configurations to improve the precision of configuration selection.

### 3.2. Integrating Meta-Learning into BOHB

Meta-learning augments BOHB by leveraging prior knowledge from previous optimization tasks to inform and enhance the current hyperparameter search. The Meta-BOHB optimizer utilizes meta-learning to learn a prior distribution over hyperparameters based on historical data from related tasks. The enhanced surrogate model p(f|λ,D,M) incorporates this prior knowledge, where $M={\left\{D^{\left(t\right)}\right\}}_{t=1}^{T}$ represents the meta-dataset from T related tasks:

(15) p(f|λ,D,M)=∫p(f|λ,θ)p(θ|M)dθ,

where θ denotes the parameters of the surrogate model learned from the meta-data M. This formulation allows Meta-BOHB to utilize knowledge from previous optimization experiences, thereby improving the surrogate model’s accuracy and accelerating the convergence to optimal hyperparameters for new tasks.

The optimization process in Meta-BOHB involves the following steps:

1. Meta-Training Phase: Utilize historical hyperparameter optimization data M to train the surrogate model, learning the prior distribution p(θ|M).
2. Initialization: Initialize the surrogate model for the current task using the learned prior.
3. Hyperparameter Suggestion: Propose new hyperparameter configurations λnext by optimizing the acquisition function a(λ|p(f|λ,D,M)).
4. Evaluation with HyperBand: Allocate computational resources using HyperBand to evaluate the suggested configurations efficiently.
5. Model Update: Update the surrogate model with the new observations D and refine the prior knowledge based on the latest results.
6. Iteration: Repeat the suggestion and evaluation steps until convergence or resource limits are reached.

The meta-learning technique slashes the time and computational power needed for hyperparameter tweaking, all while enhancing the model’s ability to perform well with previously unencountered data.

### 3.3. Application of Meta-BOHB in Rolling BearingFault Diagnosis

In this research, we implement the Meta-BOHB optimizer for rolling bearing fault diagnosis, concentrating on refining the hyperparameters of neural networks employed in fault detection. The incorporation of Meta-BOHB seeks to improve hyperparameter optimization efficiency, allowing neural networks to attain enhanced diagnostic accuracy while minimizing computational overhead. This methodology proves especially innovative for bearing fault diagnosis, where real-time implementations requiring balance between model sophistication and resource utilization remain critical [35,36].

Through Meta-BOHB implementation, the hyperparameter optimization procedure becomes more streamlined and reliable. The optimizer utilizes historical optimization data to direct the search strategy, resulting in accelerated convergence and superior model performance. This proves particularly advantageous in industrial settings where processing power and temporal constraints exist, and immediate fault diagnosis remains vital for sustaining operational effectiveness and avoiding financial impacts.

Furthermore, Meta-BOHB’s ability to generalize from previous tasks allows it to adapt to varying operational conditions and different types of bearings, enhancing the fault diagnosis model’s robustness and reliability. The enhanced diagnostic accuracy achieved through Meta-BOHB contributes to more effective health monitoring and maintenance strategies in industrial applications, ultimately improving machinery reliability and reducing economic losses from unexpected bearing failures.

## 4. Rolling Bearing Fault Diagnosis Model

**Figure 2:**
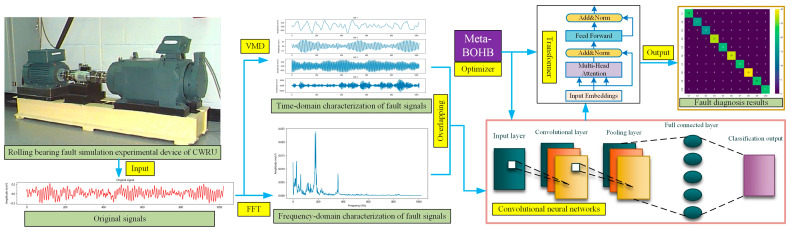
The structure of the fault diagnosis model.

Figure 2 clearly depicts the proposed rolling bearing fault diagnosis framework, offering a detailed overview of the complete methodology, spanning from initial data collection through final fault classification. The procedure begins with data acquisition from a rolling bearing fault simulation platform, such as the system established at Case Western Reserve University (CWRU). This platform generates raw vibration signals that serve as the basis for the diagnostic framework.

Following signal acquisition, the data enters a signal processing stage, which divides into two parallel pathways: one dedicated to temporal analysis and another to spectral examination. In the initial pathway, VMD methodology is employed to decompose the signal into separate temporal components. VMD extracts distinct IMFs from the signal, thereby enhancing pattern recognition capabilities for various fault categories. In the secondary pathway, FFT is implemented to convert temporal signals into spectral representations. This transformation highlights the frequency characteristics of fault signatures, facilitating the identification of spectral patterns characteristic of bearing defects.

The outputs from VMD and FFT are then combined to create an overlapping time-frequency domain representation, capturing both temporal and spectral information. This comprehensive time-frequency representation enhances the model’s capability to detect complex fault features and improves diagnostic accuracy.

The integrated time-frequency representations are processed through a CNN architecture for feature extraction and diagnostic classification. The network’s initial layer receives the preprocessed signals, while subsequent convolutional and pooling operations identify local patterns, facilitating the recognition of distinctive characteristics corresponding to various fault categories. Subsequently, a Transformer architecture is utilized to model extended temporal relationships in the data, leveraging self-attention mechanisms to emphasize critical correlations throughout the signal progression. The Transformer architecture enhances the CNN’s capabilities by incorporating global contextual information, thereby improving the system’s resilience to fluctuations in operating conditions.

The model’s hyperparameters are optimized using Meta-BOHB. Meta-BOHB leverages prior optimization experiences to dynamically search for optimal hyperparameters for both CNN and Transformer components, expediting convergence and improving the model’s efficiency. This optimization ensures that the network configuration is tailored for precise fault diagnosis with minimal computational cost.

**Figure 3:**
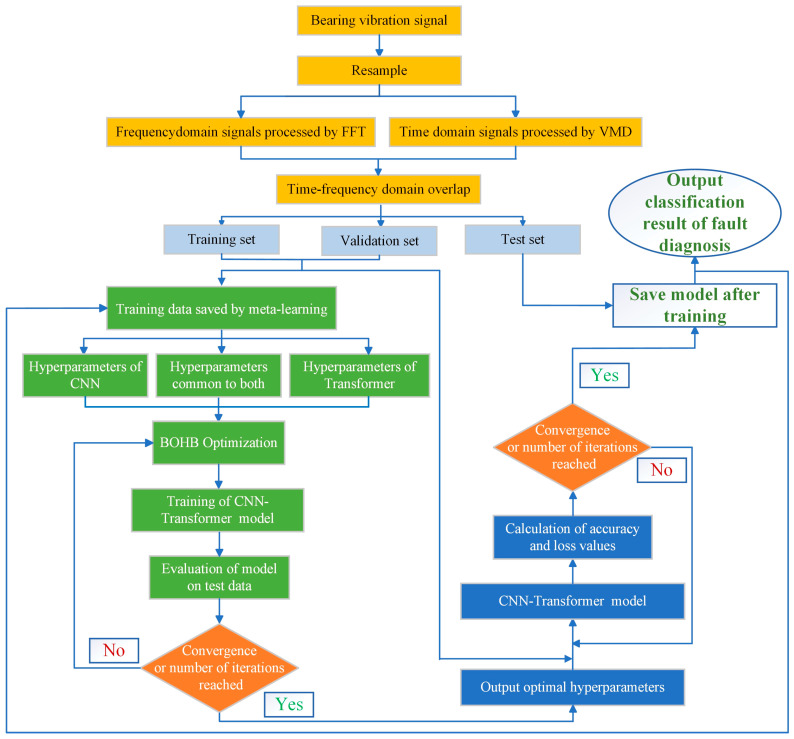
The workflow diagram of the fault diagnosis process.

Finally, after training and tuning, the model produces a fault classification output through a fully connected layer with a Softmax activation function. The output provides the specific fault type detected in the bearing, as shown in the fault diagnosis results matrix on the far right of Figure 2. This workflow, from signal acquisition to classification output, demonstrates how the integration of VMD, FFT, CNN, Transformer, and Meta-BOHB optimization enables accurate and efficient diagnosis of rolling bearing faults. The detailed process flow is illustrated in Figure 3.

## 5. Experimental Results and Analysis

### 5.1. Experimental Dataset

The experimental analysis in this research employed vibration data obtained from the publicly available bearing database provided by CWRU, United States, Mechanical Fault Prevention Technology (MFPT) [37], and Paderborn University (PU) [38] bearing dataset. The CWRU database comprises vibration measurements collected across four distinct operating states: healthy baseline, outer raceway defect, inner raceway defect, and ball bearing defect. In addition to the healthy state, each defect category includes three severity grades, resulting in ten distinct diagnostic categories. Table 1 provides detailed specifications of the investigated fault conditions.

**Figure 4:**
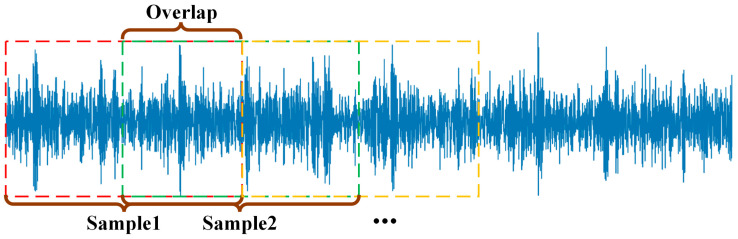
Signal segmentation.

Due to the limited quantity of samples in the original CWRU database, a comprehensive evaluation of the parallel computational capabilities of the developed diagnostic approach presented challenges. To address this limitation, an overlapping segmentation strategy was implemented for data augmentation of the vibration recordings, as illustrated in Figure 4.

Specifically, a total of 119,808 data points were utilized. Each sample consisted of 1024 data points, and adjacent segments were overlapped by 512 data points. This sliding-window approach effectively expanded the number of samples available for training and evaluation.

The MFPT dataset comprises vibration signals acquired from rolling-element bearings across various fault conditions—including inner and outer race faults with defect dimensions spanning 0.007 to 0.021 inches—and diverse operational scenarios, featuring mechanical loads from 0 to 3 horsepower. The signals are recorded at 97,656 Hz and segmented into 1 s intervals through a sliding window approach. The processed test set contains 300 samples per class for normal, inner race fault, and outer race fault states.

The PU dataset, an additional benchmark dataset established by Paderborn University, encompasses 32 bearing assemblies representing healthy, artificially induced damage, and naturally deteriorated bearings subjected to accelerated lifetime testing. Vibration data were collected using triaxial accelerometers on a test rig with a 1.5 kW motor and programmable load module (0–1 kN). To reflect real industrial variability, data were acquired under multiple lubrication conditions (normal, insufficient, contaminated), rotational speeds ranging from 1000 to 2000 RPM, and ambient temperatures from 25 °C to 80 °C. The raw signals are preprocessed with a sliding window of 2048 points and 50% overlap, yielding 200 samples per fault type. Four representative fault classes are considered: Healthy (Normal), Inner Race Fault, Outer Race Fault, and combined Inner + Outer Race Faults. The latter represents a complex real-world degradation scenario where multiple failure modes coexist.

Crucially, to prevent data leakage and ensure the strict independence of the test set, the data splitting protocol was meticulously designed. The entire continuous vibration signal for each bearing condition was first divided into non-overlapping chunks allocated for training, validation, and test sets. Only after this initial split were the 50% overlapping segments generated independently within each set. This protocol guarantees that no segment in the training or validation sets shares its source data with any segment in the test set.

### 5.2. Data Preprocessing Based on Time-Frequency Domain Analysis

Since using time-domain signals directly for fault classification leads to limited performance, this paper proposes a method that integrates both time-domain and frequency-domain features for comprehensive feature extraction. The time-domain signal is processed via VMD to extract characteristic parameters, while frequency-domain features are obtained from the spectrum generated through FFT.

**Figure 5:**
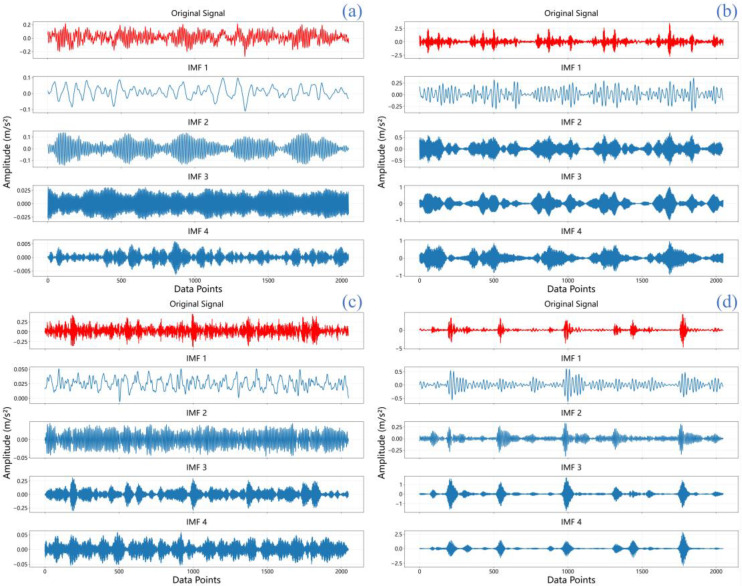
VMD processing results of bearings under 0.021-inch fault conditions: (**a**) Normal condition; (**b**) Inner race fault; (**c**) Ball fault; (**d**) Outer race fault.

In this research, raw vibration signals undergo processing through VMD and are separated into four IMFs for subsequent examination. These IMFs (IMF1-IMF4) represent distinct oscillatory modes extracted from the original signal, each capturing different frequency components and temporal characteristics that are crucial for fault feature identification. As shown in Figure 5, IMF1 typically contains the highest frequency components and often captures noise and high-frequency impacts, while subsequent IMFs (IMF2-IMF4) progressively capture lower frequency oscillations that are more closely related to bearing fault characteristics. This hierarchical decomposition enables the separation of fault-related features from background noise and other interference. The vibration data derive from the CWRU bearing dataset [37], where the initial 2048 data points are chosen as sample data encompassing both healthy and fault states. Figure 5 presents the temporal waveforms for four bearing scenarios: (a) normal, (b) inner race fault, (c) ball fault, and (d) outer race fault. Each scenario’s original signal is decomposed via VMD into four components.

The IMFs capture distinct oscillatory components embedded in the original signal, offering valuable insight into the vibration characteristics specific to each condition. Through the analysis of IMFs, distinctive vibration features specific to each bearing condition can be identified, which play a crucial role in differentiating between normal and faulty states. Each IMF captures a particular oscillatory mode embedded in the original signal, providing a more informative basis for enhanced fault diagnosis and condition monitoring.

**Figure 6:**
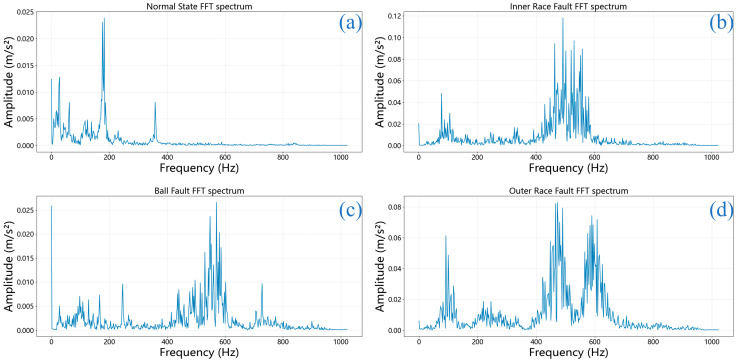
Frequency-domain characteristics of bearing signals under 0.021-inch fault conditions: (**a**) Normal condition; (**b**) Inner race fault; (**c**) Ball fault; (**d**) Outer race fault.

Simultaneously, FFT was applied to extract spectral characteristics from the original signals. Figure 6 displays the frequency-domain representations of bearing signals under a 0.021-inch fault severity for all four conditions mentioned previously.

The FFT spectra reveal significant concentration of vibration energy in the 400–600 Hz range for faulty bearings (Figure 6b–d), which is consistent with characteristic bearing fault frequencies and their harmonics under the specific rotational speed of the CWRU test rig. This frequency band likely represents resonant frequencies excited by periodic impact forces generated by bearing faults, providing valuable frequency-domain signatures for fault identification [37]. The FFT spectra reveal how the vibration energy varies across different frequencies, assisting in the identification of potential harmonics and irregularities within the vibration data. Differences in the frequency spectra across fault conditions can provide valuable insights for fault identification and diagnosis, enhancing our understanding of the bearing’s operational state and aiding in more effective monitoring.

Following the VMD and FFT processing, the resulting time-frequency representations from both methods were combined to form a comprehensive dataset. This dataset was subsequently divided into training, validation, and testing subsets at a ratio of 7:2:1, preparing it for feature extraction using the BOHB–CNN–Transformer network.

### 5.3. Simulation Result of the Proposed Method

Experimental simulations were performed on a computational platform equipped with an Intel i7-12800HX processor and an NVIDIA RTX 4060 Laptop GPU. The operational platform utilized Windows 11, while model construction, training, and assessment were executed in Python 3.11 employing the PyTorch framework.

To rigorously evaluate the model’s performance and ensure statistical robustness, we employed a 5-fold cross-validation strategy. The proposed model demonstrated exceptional and consistent performance. On the CWRU dataset, it achieved a mean classification accuracy of 99.91% ± 0.08%, confirming its robust capability in identifying all fault types and health conditions for rolling bearing diagnosis.

**Figure 7:**
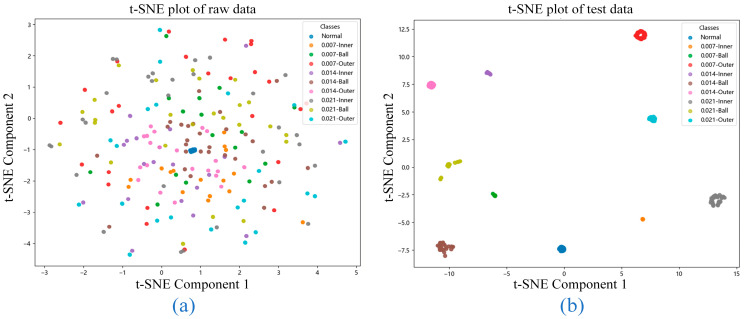
t-SNE visualizations of data distribution and classification performance: (**a**) t-SNE plot of raw data; (**b**) t-SNE plot of test data.

Figure 7 illustrates the t-distributed Stochastic Neighbor Embedding (t-SNE) visualizations that provide a comprehensive overview of both the raw data distribution and the model’s classification performance. Figure 7a shows the t-SNE plot of the raw data, where partial overlap between fault categories is observed. This overlap suggests that the dataset contains non-trivial complexities, making it challenging to distinguish certain fault types through direct inspection. The lack of clear separation among clusters indicates that additional feature extraction or dimensionality reduction is essential to achieve accurate fault identification.

In contrast, Figure 7b displays the t-SNE visualization of the test set after processing by the proposed model. Here, each fault category forms a compact and well-separated cluster with clearly defined boundaries and minimal inter-class overlap. This demonstrates the model’s success in learning highly discriminative features that enhance both inter-class separation and intra-class consistency. Notably, the model correctly classified every sample across all fault categories in the test set, confirming its comprehensive capability to handle diverse and complex fault patterns.

**Figure 8:**
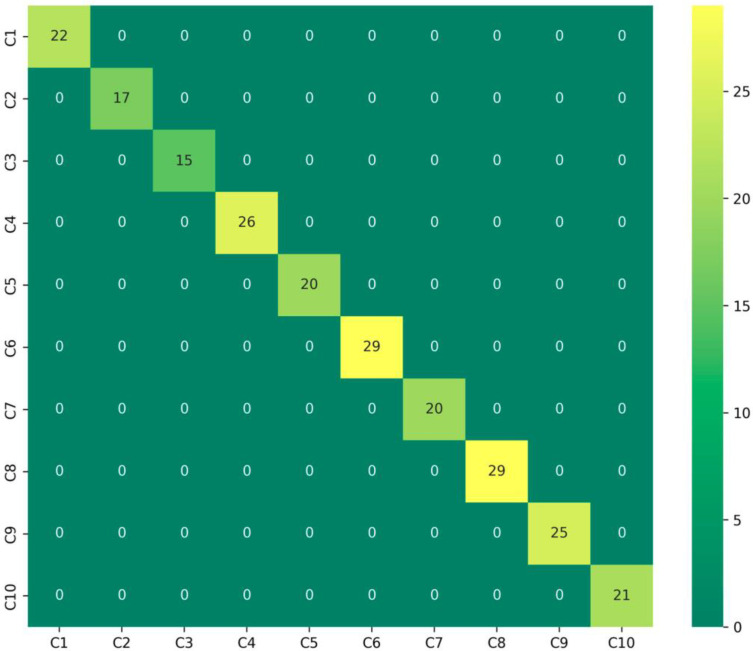
Confusion matrix of CWRU test set.

This qualitative assessment of feature separability is supported by quantitative results; the corresponding confusion matrix for the CWRU dataset (Figure 8) confirms a perfect classification outcome, with all test samples being correctly classified across every fault category. This validates the model’s comprehensive capability to handle diverse and complex fault patterns.

These t-SNE visualizations demonstrate that the model effectively handles the original data’s complexity while attaining superior classification performance through capturing detailed fault-specific characteristics. The effective separation of all fault categories validates the model’s reliability and applicability for practical fault diagnosis implementations in industrial environments.

### 5.4. Ablation Experiment

To comprehensively substantiate the role of the VMD-FFT module within the overall architecture and verify its generalizability across different bearing fault scenarios, we designed an extensive ablation study encompassing all three datasets (CWRU, MFPT, and PU). Specifically, while retaining the BOHB–CNN–Transformer structure intact, we systematically ablated the VMD and FFT preprocessing components. The classification performance of these degraded models was then rigorously evaluated and compared using 5-fold cross-validation, providing explicit and generalizable evidence for the contribution of each module.

For model performance evaluation, two primary metrics are commonly employed: accuracy and loss. Accuracy, representing the ratio of accurate predictions generated by the model, is mathematically defined as:

(16) $$Accuracy=\frac{TP+TN}{TP+TN+FP+FN},$$

where TP (True Positives) and TN (True Negatives) denote accurately classified samples, whereas FP (False Positives) and FN (False Negatives) signify misclassified instances. Conversely, loss measures the discrepancy between predicted and ground truth values, directing the optimization procedure throughout training. For this investigation, we utilized the Cross Entropy Loss function, formulated as:

(17) $$Loss=-\frac{1}{N}\sum_{i=1}^{N}\sum_{j=1}^{C}y_{ij}\log(p_{ij})$$

where N represents the total sample count, C denotes the category quantity, $y_{ij}$ serves as the binary indicator (0 or 1) when category label j represents the accurate classification of sample i, and $p_{ij}$ indicates the estimated likelihood of sample i being assigned to category j. Such evaluation measures offer thorough insights into the diagnostic system’s performance for bearing defect identification.

The results, summarized in Table 2, reveal a consistent performance hierarchy across all three datasets. The model using only VMD preprocessing achieved respectable accuracy, ranging from 94.18% on the more complex PU dataset to 96.82% on CWRU. The variant using only FFT consistently outperformed the VMD-only model, with accuracies between 95.23% and 97.95%. However, the complete model, incorporating both VMD and FFT, significantly surpassed all ablated versions. It reached near-perfect accuracy of 99.91% on CWRU and maintained superior performance of 99.05% and 98.75% on the MFPT and PU datasets, respectively. Notably, the full model also exhibited the smallest standard deviation in all cases, indicating more stable and reliable predictions.

**Figure 9:**
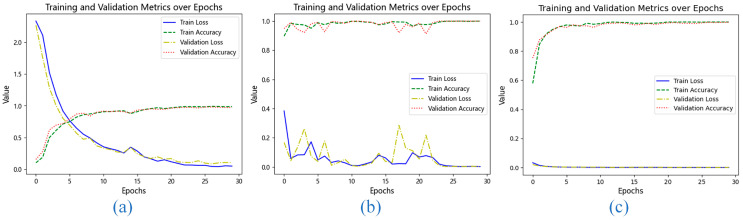
Performance comparison of diagnostic models: (**a**) Diagnostic results using only the VMD module; (**b**) Diagnostic results using only the FFT module; (**c**) Diagnostic results using the combination of VMD and FFT modules.

To gain further insight into the training behavior, we examined the loss curves and accuracy progression on the CWRU dataset, as shown in Figure 9. When only VMD was used (Figure 9a), the model converged slowly, suggesting that time-domain features alone are insufficient for efficient learning. The FFT-only configuration (Figure 9b) led to unstable training with significant fluctuations in validation loss, indicating that pure frequency-domain information, while useful, lacks the temporal precision needed for consistent feature extraction.

In contrast, the combined VMD-FFT approach (Figure 9c) resulted in a smooth and rapid decline in both training and validation loss, with accuracy stabilizing at a high level early in the training process. This demonstrates that the two preprocessing techniques are complementary: VMD provides fine-grained temporal decomposition, while FFT offers a global frequency perspective. Their integration creates a richer and more discriminative feature set, which is crucial for achieving high-precision fault diagnosis.

In summary, the ablation study confirms that the VMD-FFT module is a critical component of our proposed model. Its consistent performance gain across diverse datasets underscores its role in providing a robust and informative feature representation that is essential for accurate bearing fault diagnosis.

### 5.5. Comparison with Other Fault Diagnosis Methods

In order to validate the performance of the BOHB–CNN–Transformer model, an extensive series of comparative evaluations was conducted using the CWRU bearing dataset. Both VMD and FFT were implemented consistently throughout data preprocessing to maintain equitable and uniform comparison among all models. Four established models were chosen for benchmarking: CNN, Transformer, CNN-BiLSTM, and CNN-BiGRU.

Initially, we developed the networks employing standard hyperparameter configurations for rolling bearing fault diagnosis, which have received extensive validation across multiple fault diagnosis studies. These evaluations enabled us to create a baseline for comparison against the BOHB-optimized model.

Throughout preliminary evaluations, the classification performance of each model was assessed. The findings revealed that while all models demonstrated competence in bearing fault diagnosis, significant performance variations were observed among them. As presented in Table 3, the CNN model attained a mean accuracy of 94.8% ± 1.2%, while the Transformer achieved 96.4% ± 1.0%. Models that incorporated temporal modeling, specifically CNN-BiLSTM and CNN-BiGRU, reached accuracies of 98.3% ± 0.7% and 97.1% ± 0.9%, respectively. The CNN–Transformer model outperformed all these architectures, attaining a superior accuracy of 98.7% ± 0.3%, thereby exceeding both the individual component models and the CNN-BiGRU baseline.

The findings demonstrate that combining diverse architectural features substantially improves diagnostic precision for fault detection. The CNN–Transformer model effectively leverages the strengths of both components: the CNN excels at extracting local, hierarchical patterns from the time-frequency input, while the Transformer’s self-attention mechanism robustly captures long-range dependencies and global context across the entire signal sequence. This synergistic combination proves more effective for modeling complex vibration signatures than the sequential processing of BiLSTM/BiGRU, especially in highlighting critical, discriminative features for fault classification.

Subsequently, we introduced the BOHB algorithm to optimize the hyperparameters of each model. BOHB combines the efficiency of Bayesian optimization with the rapid convergence of Hyperband, allowing for the exploration of a wide hyperparameter space in a relatively short time to identify the optimal parameter combinations. Through this optimization, we aim to enhance the classification performance of the models, further validating the effectiveness and necessity of BOHB for deep learning models.

The hyperparameter search space for each model is detailed in Table 4, encompassing common parameters—including learning rate, batch size, and dropout rate—along with architecture-specific parameters. For instance, the CNN model’s search space incorporates the quantity of convolutional layers, filters, and hidden layers, whereas the Transformer includes the count of attention heads and layers.

By systematically exploring these hyperparameter spaces, BOHB aims to maximize the fault diagnosis accuracy of the models while minimizing overfitting and computational costs. The results obtained from these experiments not only demonstrate the effectiveness of BOHB in optimizing complex deep learning models but also highlight the comparative advantages of the BOHB–CNN–Transformer model in terms of diagnostic accuracy and robustness under varying operational conditions.

After applying BOHB to optimize the hyperparameters of each model, we observed significant improvements in performance across all models, further validating the effectiveness of this optimization technique. The BOHB algorithm’s ability to efficiently explore the hyperparameter space led to more refined models that excel in the task of bearing fault diagnosis.

**Figure 10:**
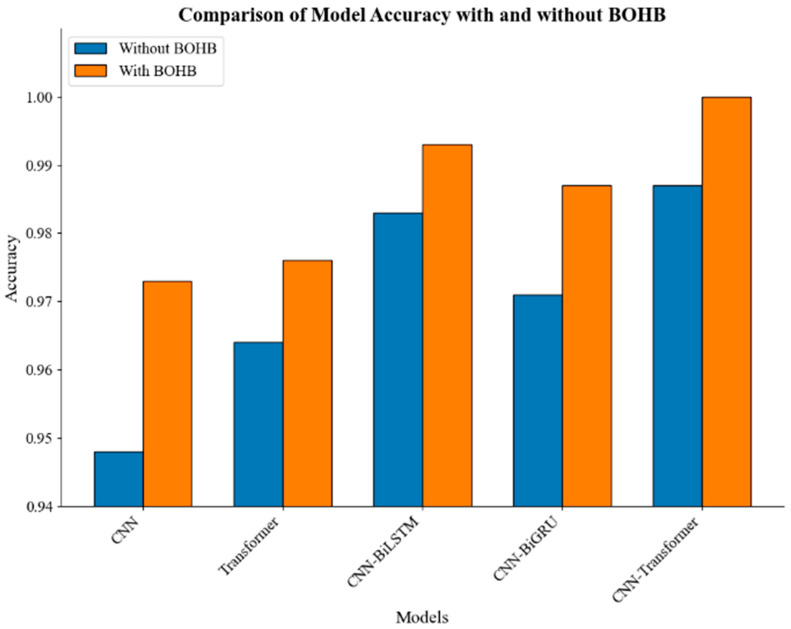
Comparison of Model Accuracy with and without BOHB.

As shown in Figure 10, all models demonstrated substantial improvements in classification accuracy after BOHB optimization. As presented in the updated results, the CNN model achieved an enhanced accuracy of 97.3% ± 0.3%, reflecting more effective tuning of convolutional layers and learning schedules. The Transformer architecture attained 97.6% ± 0.3%, indicating refined attention head configuration and layer stacking. The CNN-BiLSTM model further strengthened its temporal modeling capacity, reaching 99.1% ± 0.2%, while the CNN-BiGRU model also showed marked improvement, achieving 98.7% ± 0.2%. Most notably, the proposed CNN–Transformer model attained a near-perfect accuracy of 99.91% ± 0.08%, significantly surpassing all baseline configurations and affirming the synergistic effect of its hybrid design when coupled with Bayesian hyperparameter optimization.

### 5.6. Comparison with Other Optimization Algorithms

To comprehensively evaluate the effectiveness of BOHB in hyperparameter optimization, we conducted a focused comparative study using the CNN–Transformer model. This analysis involved comparing BOHB with three other widely recognized optimization algorithms: BO, HB, and the Sparrow Search Algorithm (SSA). The key evaluation metrics were the best loss value achieved during the optimization process and the total time required to reach this optimal solution.

The results from our experiments, as shown in Table 5, clearly demonstrate the superior performance of BOHB. BOHB achieved the lowest loss value of 0.04219011, surpassing the other methods in its ability to tune the CNN–Transformer model finely. In comparison:

(1) BO, known for its thorough exploration of the hyperparameter space, achieved a best loss value of 0.04531234. While effective in reducing the loss, BO required more time to converge, reflecting its more exhaustive approach to hyperparameter tuning.
(2) HB performed the optimization process faster than BO and SSA, completing it in less time but at the expense of achieving a slightly higher loss value of 0.04876542. HB’s efficiency in quickly identifying promising hyperparameter configurations is evident, though it sometimes sacrifices the precision needed to achieve the lowest possible loss.
(3) SSA, while useful for exploring large and complex search spaces, resulted in the highest loss among the algorithms, with a best value of 0.05282499. The stochastic nature of SSA led to higher variance in outcomes, requiring more iterations to stabilize and leading to suboptimal configurations compared to BO and HB.

In the comparative evaluation of hyperparameter optimization methods, BOHB emerged as a highly efficient and effective approach, completing the optimization process in 12 min and 11 s. This underscores BOHB’s ability to balance thorough exploration of the hyperparameter space with rapid convergence, making it particularly well-suited for complex models like CNN–Transformer.

In contrast, BO, known for its exhaustive search capabilities, required 18 min and 30 s to achieve its results, reflecting the intensive nature of its exploration process. HB delivered the fastest optimization time, completing the process in just 10 min and 7 s; however, this speed often comes at the expense of fine-tuning, as HB’s aggressive resource allocation may prematurely discard potentially optimal configurations. The SSA, although effective in exploring large search spaces, was the slowest, taking 24 min and 23 s to converge, highlighting SSA’s iterative and stochastic nature, which can lead to longer optimization times.

This demonstrates that our approach introduces only a marginal increase in search complexity over the simplest HB, but this minor cost is overwhelmingly justified by a dramatic boost in performance, making it a highly efficient and effective solution for automated hyperparameter tuning.

Overall, these findings highlight BOHB’s superior ability to deliver optimal hyperparameter settings in a timely manner, making it the preferred method for scenarios where both accuracy and efficiency are critical. BOHB’s balance of precision and speed positions it as a powerful tool in the toolkit of machine learning practitioners, particularly in applications requiring complex model tuning.

### 5.7. The Role of Meta-Learning in the BOHB Optimizer

In this subsection, we replace the original dataset with two widely adopted fault diagnosis benchmarks—the MFPT [37] and the PU [38] bearing dataset—to rigorously evaluate the meta-generalization capability of BOHB across different mechanical fault scenarios.

The performance of the optimizers—Meta-BOHB, standard BOHB, and BO—is evaluated based on two criteria:

1. Iterations to convergence, which measures adaptation speed.
2. Best loss value, reflecting the quality of the hyperparameter configuration.

**Figure 11:**
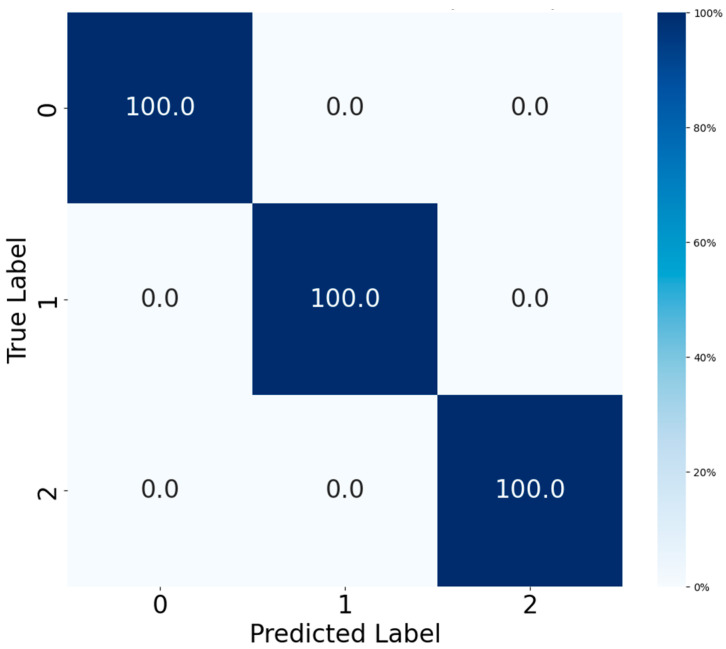
Confusion matrix of MFPT test set.

**Figure 12:**
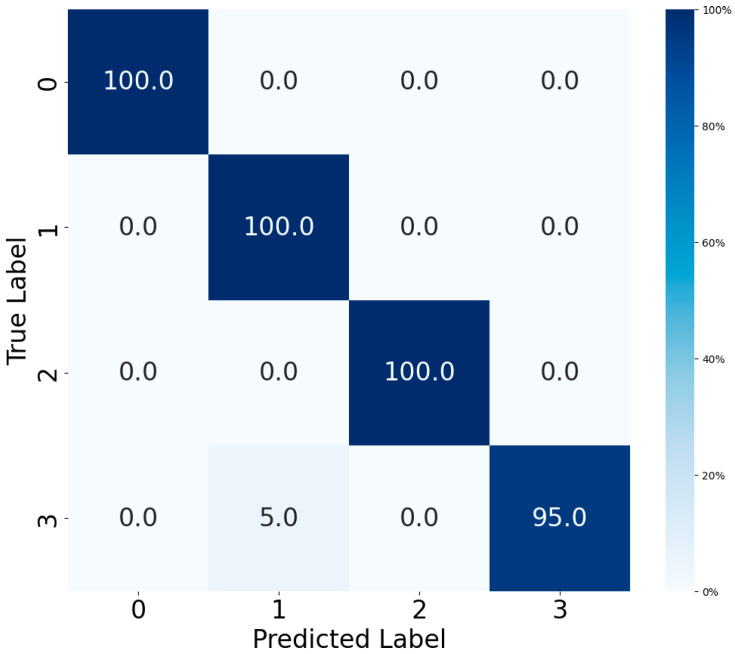
Confusion matrix of PU test set.

Figure 11 and Figure 12 present the confusion matrices of the fault diagnosis model optimized by Meta-BOHB on both the MFPT and PU datasets, illustrating its exceptional classification performance. On the MFPT dataset, the model achieves 100% accuracy across all three fault types (normal, inner race fault, and outer race fault), demonstrating perfect separability under controlled fault conditions. On the PU dataset, the model also achieves high performance with an overall accuracy of 98.75%, though one sample of inner + outer race fault is misclassified as inner race fault due to the high similarity in vibration characteristics.

As summarized in Table 6, the proposed Meta-BOHB demonstrates superior optimization efficiency and performance on both the MFPT and PU datasets compared to baseline methods. On MFPT, Meta-BOHB converges in just 5 iterations with a loss value of 0.0418, while BOHB and BO require 13 and 18 iterations with loss values of 0.0421 and 0.0450, respectively. On the more complex PU dataset, Meta-BOHB achieves convergence within 7 iterations with a loss value of 0.0432, outperforming both standard BOHB (12 iterations, loss: 0.0448) and BO (17 iterations, loss: 0.0473). The improved convergence is attributed to the meta-learning framework, which leverages knowledge from historical optimization tasks to effectively initialize the search process. This reduces redundant exploration and prioritizes hyperparameter regions associated with robust performance in fault diagnosis applications.

## 6. Conclusions

This study has successfully developed and validated a novel intelligent fault diagnosis framework for rolling bearings that integrates advanced signal processing with an optimized hybrid deep learning architecture. The proposed model effectively addresses key challenges in the field, including the handling of non-stationary vibration signals, automatic feature extraction, and the computationally expensive process of hyperparameter tuning.

The core of our methodology lies in the synergistic combination of VMD and FFT for time-frequency domain analysis, which provides a rich and discriminative feature representation from raw vibration data. This preprocessed information is then fed into a hybrid CNN–Transformer network, capitalizing on the complementary strengths of CNNs in local feature extraction and Transformers in capturing long-range temporal dependencies. To further enhance the framework’s practicality and performance, we introduced the Meta-BOHB optimizer, which significantly accelerates the hyperparameter configuration process by leveraging knowledge from prior tasks.

Extensive experimental results on the CWRU dataset, rigorously evaluated using 5-fold cross-validation, demonstrate the exceptional capability of our model, achieving a remarkable mean accuracy of 99.91% with minimal standard deviation. The superiority of the proposed BOHB–CNN–Transformer model was further confirmed through comprehensive comparative studies, where it consistently outperformed other established deep learning architectures. More importantly, the model exhibits outstanding generalization ability, as validated on the MFPT and PU datasets, achieving high accuracy with rapid convergence. The critical role of each component was unequivocally verified through ablation studies conducted across all three datasets.

In summary, the proposed framework offers a robust, accurate, and efficient solution for rolling bearing fault diagnosis. Its strong performance and adaptability under diverse operating conditions highlight its significant potential for enhancing predictive maintenance strategies, improving machinery reliability, and reducing operational costs in industrial applications.

Future work will focus on exploring several promising directions to bridge the gap between laboratory research and real-world deployment. These include: (1) extending the framework’s capability to diagnose compound faults and faults under time-varying operating conditions; (2) investigating the integration of transfer learning techniques to facilitate domain adaptation with minimal labeled data; (3) exploring the deployment of the optimized model on edge computing devices for real-time, in situ fault diagnosis in challenging industrial environments.

## Figures and Tables

**Table 1:** Fault status information.

Fault Type	Fault Diameter (in.)	Number of Samples	Label
Inner Race Fault	0.007	233	1
	0.014	233	2
	0.021	233	3
Outer Race Fault	0.007	233	4
	0.014	233	5
	0.021	233	6
Ball Fault	0.007	233	7
	0.014	233	8
	0.021	233	9
Normal	None	233	10

**Table 2:** Ablation study results of the VMD-FFT module across multiple datasets (Mean Accuracy ± Standard Deviation, %).

Model Configuration	CWRU	MFPT	PU
VMD only	96.82 ± 0.31	95.47 ± 0.45	94.18 ± 0.62
FFT only	97.95 ± 0.41	96.88 ± 0.52	95.23 ± 0.71
VMD + FFT (Proposed)	99.91 ± 0.08	99.05 ± 0.15	98.75 ± 0.24

**Table 3:** Classification Accuracy of Different Models without BOHB.

Model	Accuracy (Mean Accuracy ± Standard Deviation, %)
CNN	94.8 ± 1.2
Transformer	96.4 ± 1.0
CNN-BiLSTM	98.3 ± 0.7
CNN-BiGRU	97.1 ± 0.9
CNN–Transformer	98.7 ± 0.3

**Table 4:** Hyperparameter search space for each model.

Model	Hyperparameter	Search Space
Common parameters	Learning Rate	[10 × 10^−5^, 10 × 10^−3^]
	Batch Size	[32, 128]
	Dropout Rate	[0.1, 0.5]
CNN	Number of Convolutional Layers	[2, 8]
	Number of Filters	[16, 256]
Transformer	Number of Attention Heads	[4, 12]
	Number of Transformer Layers	[2, 8]
CNN + LSTM	Number of LSTM Units	[50, 150]
	Number of Convolutional Layers	[2, 8]
CNN + GRU	Number of GRU Units	[50, 150]
	Number of Convolutional Layers	[2, 8]

**Table 5:** Performance comparison of optimization algorithms.

Optimization Method	Best Loss Value	Time Taken
BOHB	0.0421	12 m 11 s
Bayesian Optimization	0.0453	18 m 30 s
Hyperband	0.0487	10 m 07 s
Sparrow Search Algorithm	0.0528	24 m 23 s

**Table 6:** Performance of optimizers on the MFPT and PU datasets.

Optimization Method	Iterations to Convergence (MFPT)	Best Loss Value (MFPT)	Iterations to Convergence (PU)	Best Loss Value (PU)
Meta BOHB	5	0.0418	7	0.0432
BOHB	13	0.0421	12	0.0448
Bayesian Optimization	18	0.0450	17	0.0473

## Data Availability

The original contributions presented in the study are included in the article; further inquiries can be directed to the corresponding author.

## Abbreviations

The following abbreviations are used in this manuscript:

ADMM	Alternating Direction Method of Multipliers
ANNs	Artificial Neural Networks
BO	Bayesian Optimization
BOHB	Bayesian Optimization and HyperBand
BPNN	Back-Propagation Neural Network
CNN	Convolutional Neural Network
CNNs	Convolutional Neural Networks
CWRU	Case Western Reserve University
DEA	Differential Evolution Algorithm
EI	Expected Improvement
EMD	Empirical Mode Decomposition
FFT	Fast Fourier Transform
GP	Gaussian Process
GWO	Gray Wolf Optimization
HB	HyperBand
IMF	Intrinsic Mode Function
MFPT	Mechanical Fault Prevention Technology
Meta-BOHB	Meta-learning-enhanced Bayesian optimization and HyperBand
PSO	Particle Swarm Optimization
PU	Paderborn University
QPSO	Quantum Particle Swarm Optimization
RNNs	Recurrent Neural Networks
SSA	Sparrow Search Algorithm
SVM	Support Vector Machine
t-SNE	t-distributed Stochastic Neighbor Embedding
TPE	Tree-structured Parzen Estimator
VMD	Variational Mode Decomposition
